# Radiation‐induced skin toxicity: prophylaxis or management?

**DOI:** 10.1002/jmrs.418

**Published:** 2020-08-21

**Authors:** Pauline Rose

**Affiliations:** ^1^ Radiation Oncology Raymond Terrace Centre Princess Alexandra Hospital Brisbane Queensland Australia

## Abstract

This editorial aims to provide context for changing skin care interventions for radiation‐induced skin toxicities over several decades.

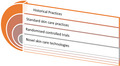

The management of radiation‐induced (RI) skin toxicities has been an enduring problem since the beginning of radiation therapy as a treatment modality. For many years, the management of this treatment side effect was in somewhat of a holding pattern of tradition and anecdote, with the burning question being ‘to wash or not to wash?’. However, experiential and research‐based knowledge and improving technologies eventually evolved to deliver a standard of care for many radiation oncology departments. Patients were advised to wash the skin gently with the aim of maintaining good skin hygiene during standard skin care practices. There are still, however, radiation treatment departments globally that advise patients not to wash the skin in the treatment area during the course of radiation therapy.^1^

The literature has shown that there are two aspects to managing irradiated skin: (i) prevention of skin damage, and then (ii) managing any damage that does occur. There has generally been a feeling of inevitability amongst radiation oncology professionals that, regardless of what is advised and utilised, there will always be a level of skin damage, especially in areas at risk such as skin folds. To this end, the aims of any radiation therapy skin care protocol have been to minimise symptoms, provide comfort and pain relief, not threaten the integrity of the treatment area, support quality of life and eliminate treatment interruptions. Avoidance of the three main irritants – mechanical, chemical and thermal – have been the basis of skin care education for patients undergoing a course of radiation therapy. A standard practice in many radiation oncology departments has been to moisturise the skin at least twice daily to prevent xerosis (dry skin), dry desquamation and pruritus. Then, if the skin reaction progresses to areas of moist desquamation, the use of a dressing such as a hydrogel is indicated, with its associated supplementary dressings. There are significant costs associated with these interventions, especially if required to support patients in the post‐radiation phase.

Cancer journals are literally awash with articles about the management of RI skin toxicities. And it’s not just radiation oncology professionals who are concerned about it – researchers include plastic surgeons, wound care nurses, radiologists and dermatologists, amongst others. Over time, researchers have trialled a great many topical skin products and dressings in order to manage this condition. Components of skin preparations have included a virtual *pot pourri* of natural ingredients such as aloe vera, pawpaw, cows’ milk, lavender oil, red, green and white tea, calendula, carmellia, goji berry and pomegranate, as well as the pharmaceutical preparations.^2^ Systematic reviews of randomised controlled trials (RCT) in the management of RI skin reactions have concluded the evidence for topical skin applications has been limited and inconclusive at best.^3^

Technologies and techniques in cancer treatment – both in radiation therapy and medical oncology – have developed exponentially over recent years. Despite this, the use of concurrent chemoradiation therapy (CRT) protocols means that skin toxicity continues to be an important problem in clinical practice, especially to sites such as head and neck, and bolus requirements continue to enhance the skin dose and thus the severity of skin reactions in areas such as chest wall.

Since 2004, barrier products have been trialled in an effort to reduce friction trauma to the skin during the course of radiation therapy. One of the earlier barrier products was a barrier film (BF). The prophylactic use of a non‐sting barrier film versus sorbolene cream was trialled in a phase II RCT in 61 post‐mastectomy women undergoing radiation therapy. The authors found a non‐significant reduction in Grade>2  dermatitis, moist desquamation rates and pruritis.^4^

In the past decade, skin care technologies have seen new players enter the market for the prevention or minimisation of RI skin toxicities. The advent of silicone‐based dressings, films and gels is potentially one of the most important interventions currently being introduced and researched. This range of protectant technologies aim to reduce trauma to the treatment site by providing a more effective barrier to mechanical damage and chemical irritants during activities of daily living (ADL), at the same time reducing the transepidermal water loss required to enhance skin healing. In 2008, a European group described a case study assessing the use of a silicone dressing throughout radiation therapy and found the intervention promising as an alternative to existing dressings at the time. Unfortunately, the dressing could not be left on during treatment due to a significant bolus effect.^5^

In 2014, Herst and colleagues reported on a phase III trial of silicone film versus aqueous cream to prevent radiodermatitis in 78 breast cancer patients in New Zealand.^6^ These authors found that overall skin reaction severity was reduced by 92% (p < 0.0001) in favour of the silicone film when used prophylactically. In this trial, the skin of each patient was divided into a lateral and medial half and each half was randomised to either silicone film or control cream. All of the cream‐treated skin patches showed some form of skin reaction that progressed to moist desquamation in 26% of patients, whereas none of the skin patches covered by silicone film progressed to moist desquamation. Next, Herst et al.,^7^ in a New Zealand ‐ Chinese collaboration, conducted the first silicone feasibility RCT in head and neck patients undergoing radiation therapy. This intra‐patient controlled trial compared silicone film with sorbolene cream in 22 New Zealand patients and Biafine cream in 11 Chinese patients. The study reported significant reductions in skin reaction severity and moist desquamation rates when used in the prophylactic setting. In 2019 a Queensland group conducted a phase III RCT of a silicone gel for prophylaxis and management of RI dermatitis in 197 head and neck cancer patients.^8^ This trial reported that a silicone gel was superior to sorbolene in preventing, delaying and reducing the severity of skin reaction during radiation therapy to the head and neck.

In this issue of *Journal of Medical Radiation Sciences* the New Zealand‐Chinese collaboration between Dr Herst and Dr Yan report and discuss results from their latest intra‐patient controlled phase II RCT. This study assessed Mepitel Film versus Biafine cream during radiation therapy in 44 Chinese head and neck cancer patients.^9^ Similar to their previous head and neck study^7^ they found that Mepitel Film, when used prophylactically, significantly decreased the severity of acute RI skin reactions by 30%, and moist desquamation rates by 41%.

The evidence to support the use of silicone‐based products has been steadily increasing through a series of clinical trials by various researchers since 2004. This latest study adds further to the body of evidence that suggests that Mepitel Film significantly decreases radiation‐induced skin reactions in both breast and head and neck cancer patients.
